# Static stretch and dynamic muscle activity induce acute similar increase in corticospinal excitability

**DOI:** 10.1371/journal.pone.0230388

**Published:** 2020-03-19

**Authors:** Jules Opplert, Christos Paizis, Athina Papitsa, Anthony J. Blazevich, Carole Cometti, Nicolas Babault

**Affiliations:** 1 INSERM UMR1093-CAPS, University of Burgundy Franche-Comté, Faculty of Sport Sciences, Dijon, France; 2 Performance Expertise Center, University of Burgundy Franche-Comté, Faculty of Sport Sciences, Dijon, France; 3 School of Medical and Health Sciences and Centre for Exercise and Sports Science Research, Edith Cowan University, Perth, Australia; Universidade Federal de Mato Grosso do Sul, BRAZIL

## Abstract

Even though the acute effects of pre-exercise static stretching and dynamic muscle activity on muscular and functional performance have been largely investigated, their effects on the corticospinal pathway are still unclear. For that reason, this study examined the acute effects of 5×20 s of static stretching, dynamic muscle activity and a control condition on spinal excitability, corticospinal excitability and plantar flexor neuromuscular properties. Fifteen volunteers were randomly tested on separate days. Transcranial magnetic stimulation was applied to investigate corticospinal excitability by recording the amplitude of the motor-evoked potential (MEP) and the duration of the cortical silent period (cSP). Peripheral nerve stimulation was applied to investigate (i) spinal excitability using the Hoffmann reflex (H_max_), and (ii) neuromuscular properties using the amplitude of the maximal M-wave (M_max_) and corresponding peak twitch torque. These measurements were performed with a background 30% of maximal voluntary isometric contraction. Finally, the maximal voluntary isometric contraction torque and the corresponding electromyography (EMG) from soleus, gastrocnemius medialis and gastrocnemius lateralis were recorded. These parameters were measured immediately before and 10 s after each conditioning activity of plantar flexors. Corticospinal excitability (MEP/M_max_) was significantly enhanced after static stretching in soleus (P = 0.001; ES = 0.54) and gastrocnemius lateralis (P<0.001; ES = 0.64), and after dynamic muscle activity in gastrocnemius lateralis (P = 0.003; ES = 0.53) only. On the other hand, spinal excitability (H_max_/M_max_), cSP duration, muscle activation (EMG/M_max_) as well as maximal voluntary and evoked torque remained unaltered after all pre-exercise interventions. These findings indicate the presence of facilitation of the corticospinal pathway without change in muscle function after both static stretching (particularly) and dynamic muscle activity.

## Introduction

Static stretching (SS) is traditionally incorporated into pre-exercise routines in rehabilitation and sporting environments [1]. It usually involves moving a limb to its end range of motion (ROM) and holding this stretched position for several seconds [2], and has been demonstrated to be an effective method of increasing ROM about a joint [3], which may also acutely impair muscle function [4]. Whilst it has been suggested that peripheral (muscular) adaptations such as reductions in musculotendinous stiffness [5] might underpin the changes in muscle function, some evidence indicates that acute changes at multiple sites within the central nervous system (supra-spinal, spinal) are more critical [4].

Active warm-up activities (such as dynamic stretching, consisting of agonist muscle contractions to move the joint through a full active ROM and stretching the antagonist muscle [6]) are also commonly implemented in pre-exercise routines. Such activities may increase joint ROM and stretch tolerance (i.e. the maximum force tolerated during stretch) as well as reduce musculotendinous stiffness [7,8]. Furthermore, dynamic stretching has been shown to increase heart rate and both core and muscle temperature [9], decrease the viscous resistance of muscles [10] and induce a transient improvement in muscular contractility [11]. Evidence also indicates that it may increase muscular coordination and proprioception [12]. Recently, it has been suggested that, during dynamic stretching, muscle-tendon stretching effects would be partly counteracted by muscle warm-up effects, due to the inherent voluntary contractions [8]. Therefore, dynamic stretching would appear more like a dynamic muscle activity (DMA) rather than just a muscle-tendon stretching [8]. Such a warm-up may therefore be favorable in the clinical environment compared to static stretching when a minimum of time is available to condition the neuromuscular system for exercise.

Nevertheless, little information exists describing the state of the corticospinal pathway after dynamic muscle activity. Previous studies that have examined stretch-induced effects have tested the pathway during the stretch, and revealed spinal and/or cortical inhibitions [13,14,15], but not in the period after stretch cessation. The limited data available do not present a clear consensus, with a complete recovery of neural inhibition [14] or rather a transient facilitation of spinal [16] and corticospinal [17] excitability being reported. Such changes are suggestive of an altered efferent neural (i.e. central) drive to the muscle [4] and also sensory afferent inputs from muscle spindles, mechanoreceptors and nociceptors [18]. It is known that mechanisms underlying changes in excitability could be mediated by a number of central and peripheral sources. For instance, it has been demonstrated that voluntary muscle activation likely increases corticospinal excitability relative to rest [19]. It has been also shown that spinal and corticospinal excitability modulation depends on the amplitude of movement [14], with inhibitory mechanisms being greater with larger amplitude movements. As these properties differ between static stretching and dynamic muscle activity, differences in neurophysiological modulations can be expected after these pre-exercise interventions.

Given the above, we have attempted to identify the acute effects of two different pre-exercise interventions (static stretching versus dynamic muscle activity) on neuromuscular properties, with a special interest on the corticospinal pathway modulations. Considering that the literature concluded on a complete recovery of neural inhibition or a transient facilitation after static stretching, and based on the evidence that the state of corticospinal pathway depends on the movement amplitude and the muscle activation, we hypothesized a greater increase in corticospinal excitability following dynamic muscle activity, mainly due to the repeated muscle activity and the inherent lower lengthening amplitude relative to static stretching.

## Materials and methods

Fifteen (mean ± standard deviation: age 23.8 ± 2.9 years; 95 height 183.3 ± 6.2 cm; body mass 84.3 ± 10.8 kg) healthy active men (7.5 ± 3.3 h of physical activity per week, such as handball, rugby and soccer), were recruited between April and July 2018, for this randomized and controlled trial which took place in the Faculty of Sport Sciences of Dijon. We excluded individuals that had neuromuscular or musculoskeletal disease, or were involved in other experiment that might be expected to affect the current study. All of participants were recruited from the Sport Sciences Faculty to ensure a good homogeneity of the subjects, whose could be considered representative of a larger recreational athletes population. They were all volunteers and gave their written consent to participate in the experiment after being informed about the study requirements. The study conformed to the standards set by the World Medical Association Declaration of Helsinki “Ethical Principles for Medical Research Involving Human Subjects” (2008) and approval was obtained from the “EST-1 committee on human research”.

### Experimental procedure

**Fig 1:**
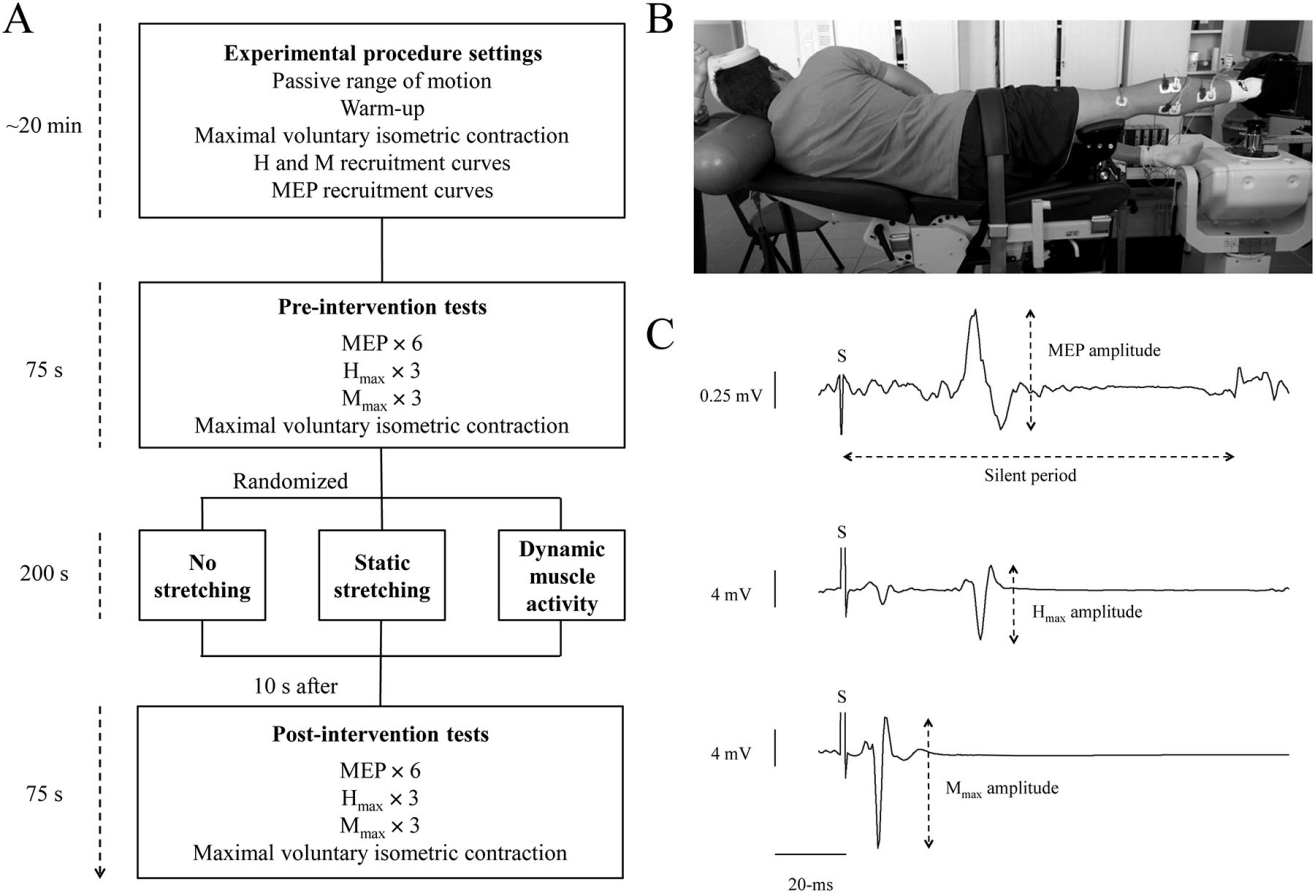
(A) Timeline of the experimental protocol showing the three (randomized) interventions. (B) Experimental setup with the subject in the neutral position. (C) A representative recording showing MEP, Hmax and Mmax electrical responses in soleus during a submaximal (30% MVIC) isometric contraction. S: Stimulation imposed.

Subjects attended the laboratory on four separate occasions to determine the effects of three conditioning interventions on spinal excitability, corticospinal excitability and neuromuscular properties of the plantar flexor muscles. The first session served as familiarization and consisted of practicing the different stretching protocols as well as the testing procedure. Three testing sessions were then completed in random order with at least 48 h between, including (a) control (CON), (b) static stretching (SS) and (c) dynamic muscle activity (DMA). Tests were performed immediately before (pre-intervention tests) and 10 s after (post-intervention tests) the conditioning protocols in order to quantify changes in spinal and corticospinal excitability (Fig 1A). As the study involved implementation of an acute intervention, limb dominance was ignored and all stretching procedures were conducted on the right plantar flexor muscles.

Electromyography (EMG) was recorded from the three plantar flexor muscles of the right leg, soleus (SOL), gastrocnemius medialis (GM) and gastrocnemius lateralis (GL) (Fig 1B). The skin was shaved and cleaned with alcohol to obtain low impedance (<5 kΩ). The EMG signals were collected by three pairs of silver-chloride electrodes (10-mm diameter) with an interelectrode distance of 2 cm. To record SOL EMG, electrodes were applied 2 cm below the intersection of the gastrocnemii over the Achilles tendon. For GM and GL, the electrodes were placed over the midbelly of the muscles. The reference electrode was fixed to the patella of the left leg. EMG signals were amplified with a bandwidth frequency ranging from 10 Hz to 5 kHz (gain = 500). EMG signals were recorded at a sampling rate of 2 kHz using the Biopac MP150 system (Biopac System, Santa Barbara, CA) and stored for analysis with the AcqKnowledge software (AcqKnowledge 4.2 for MP systems, Biopac System, Santa Barbara, CA).

Stretching and testing were performed on an isokinetic dynamometer (Biodex System 4, BIODEX Corporation, Shirley NY, USA). Subjects were positioned laterally (on their left side) to avoid gravity influence of the footplate (Fig 1B). Indeed, with this position, plantar- and dorsiflexions were performed in the horizontal axis rather than usually used vertical [20]. The left leg was flexed (about 90°) for comfort and the right leg fully extended (0°) to ensure that the triceps surae were placed under significant stretch and contributed significantly to plantar flexor torque [21]. To maintain this position, the right knee was fixed to a dynamometer support. The foot was securely attached to the footplate of the dynamometer. To minimize heel displacements, the foot was first positioned and fastened inside a shoe (adapted to subjects’ size and fixed by the sole to the dynamometer footplate) and then firmly attached to the footplate with straps. The lateral malleolus was aligned to the center of rotation of the dynamometer. From here, subjects were in a fixed position attached to the dynamometer for ~25 min (Fig 1A). The passive maximal range of motion was first determined by the experimenter starting from a maximal plantar flexion, then slowly stretching the plantar flexor muscles until the point of maximal tolerated discomfort and returned immediately to a neutral position (0° = sole of the foot perpendicular to the leg). Then, and after a short warm-up composed of ten incremental submaximal voluntary contractions, a maximal voluntary isometric contraction (MVIC) was performed to determine the level of muscular activation during stimulations (see below). Before tests were initiated, H-reflex and M-wave recruitment curve was established and the active motor threshold was determined while the subjects maintained a contraction of 30% MVIC in the neutral position [22].

Neuromuscular electrical stimulation techniques were used to investigate spinal excitability through H-reflex and M-wave measurements and neuromuscular properties using the amplitude of the maximal M-wave and corresponding peak twitch torque (PTT). The posterior tibial nerve was stimulated with single rectangular pulses (1 ms) using a Digitimer stimulator (DS7A, Hertfordshire, UK) during isometric plantar flexor contractions at 30% MVIC with the ankle in the neutral position (Fig 1B). The cathode (10 mm diameter) was placed in the popliteal fossa and the anode (5 × 10 cm) was placed on the patella on the anterior surface of the knee. The optimum stimulation site to obtain SOL H-reflex was located by a hand-held stimulation probe [23,24], then the stimulation electrode was strapped to this site. The recruitment curve was built by increasing the stimulation intensity (i) from 0 mA to SOL H_max_, then (ii) until SOL M_max_, using respectively 2 and 5 mA increments. Subjects were asked to reach a plateau of 30% MVIC and maintain the force for 1 s after each stimulation. Two stimulations were delivered at each intensity with a 5-s interval between stimuli.

Transcranial magnetic stimulation was then used to evoke MEPs in order to assess corticospinal excitability. A double-cone coil connected to a magnetic stimulator (Magstim 2002, Magstim, Whitland, Dyfed, UK) was positioned over the primary motor cortex at the area of the homunculus corresponding to the triceps surae muscle approximately 10 mm posterior and lateral to the vertex of the subjects’ head [23] (Fig 1B). The stimulation site providing the greatest amplitude response for SOL was then identified [23,24] by delivering pulses between 50 and 65% of the maximal stimulator output during isometric contractions maintained at 30% MVIC in the neutral position. Once the optimal site was determined, it was marked with adhesive tape to ensure a constant positioning of the coil throughout the experiment. Active motor threshold, defined as the minimal intensity at which at least three of four evoked responses were detected, was then identified [25] by increasing the stimulus intensity from 30% in steps of 1 to 5% of the maximal stimulator output. Only four stimulations were delivered at each intensity to minimize fatigue, with a 5-s interval between stimuli.

### Testing procedure

Six MEPs were evoked at an intensity corresponding to 130% of the ac**t**ive motor threshold [25], before the predetermined H_max_ and M_max_ stimulation intensities were applied three times each. A 5-s interval was set between stimuli and subjects were asked to reach a plateau of 30% MVIC before each MEP, H_max_ and M_max_ stimulation and to hold it for 1 s after. Finally, a single 5-s maximal voluntary isometric contraction was performed. The testing procedure (total duration = ~75 s) was conducted immediately before (pre-intervention tests) and 10 s after (post-intervention tests) the conditioning activity, in the presented order.

### Conditioning activities

All conditioning activities were time matched to permit direct comparison. During the control condition, the subject’s ankle was held in the neutral position and they were instructed to relax for a duration corresponding to the stretching protocol (200 s). The static stretching intervention included five sets of static stretching. The subject’s ankle was passively rotated by the isokinetic dynamometer until the maximal dorsiflexion previously individually determined at 5° · s^-1^; a slow angular velocity was set in order to avoid the myotatic reflex [26]. For consistency, the range of motion used corresponded to the final 25° until the maximal dorsiflexion. Each set of static stretching took 20 s, including 5 s of lengthening and a 15-s hold before the ankle was released immediately to the start position at 60° · s^-1^ in readiness for the next stretch (20-s between-stretch rest). Thus, the total stretch procedure lasted for ~200 s (depending on the time to move the ankle to the individual final 25°). The subjects were instructed to relax during the stretching and not to offer any resistance to the dynamometer. To ensure stretching procedure was passive, the electromyography of the three triceps surae muscles was collected during static stretching and normalized to the electromyography recorded during maximal voluntary isometric contraction. Subject’s data were excluded if normalized EMG was greater than 5% of the EMG recorded during MVIC [27]. For the dynamic muscle activity condition, the subjects were instructed to move their right foot towards maximal dorsiflexion as fast as possible against the lowest resistance (0.5N) provided by the mechanical inertia of the isokinetic dynamometer, and to then extend their foot back towards maximal plantar flexion at a self-regulated pace that permitted a dorsi-plantar flexion cycle frequency of 1 Hz, set by a metronome. Each set of dynamic muscle activity took 20 s and included 20 cycles (20-s between-set rest), providing a light dynamic muscle warm-up whilst moving the joint through a full active ROM, i.e. ~79°.

### Data analysis

The peak-to-peak MEP, H_max_ and M_max_ amplitudes were measured during submaximal isometric contractions (e.g. Fig 1C). The average of all MEP and H_max_ amplitudes were normalized to the average M_max_ amplitude, providing a measure of spinal (H_max_/M_max_) and corticospinal (MEP/M_max_) excitability, respectively [22]. EMG during contractions was quantified using root mean square values of the EMG signal in the 200-ms period prior to stimulation [28] for post-hoc examination of the reliability of contractions. The duration of the cortical silent period was taken as the time interval from the stimulus artifact to the return of EMG (Fig 1C). The end of the cortical silent period was established when the corresponding EMG reached a value within two standard deviations of the EMG signal recorded during the 200-ms window immediately before the stimulation [22]. The mechanical responses resulting from stimulations to evoke M_max_ at 30% MVIC were used to calculate the peak twitch torque. Additionally, maximal voluntary isometric torque (MVIT) was recorded from the MVIC. Finally, EMG was quantified with root mean square values of the EMG signal over a 200-ms period and normalized to the M-wave amplitude measured before the MVIC (EMG/M_max_) [29]. For SOL, GM and GL, (i) MEP, H_max_ and M_max_ amplitudes (as EMG values), (ii) MEP/M_max_, H_max_/M_max_ and EMG/M_max_ ratios, and (iii) cortical silent period durations, were calculated. Peak torque developed by plantar flexors muscles during each plantar flexion movements of DMA was also recorded, averaged and reported to the MVIT.

### Statistical analysis

For all variables, data distribution was quantified by the Shapiro-Wilk test. Because all variables were normally distributed, a two-way (conditioning activity × time) analysis of variance (ANOVA) with repeated measures was conducted on absolute values. Conditioning activity corresponded to CON, SS and DMA. Time corresponded to pre- and post-intervention tests. When significant main effects or interactions were present, Bonferroni correction was used as a post-hoc test. Statistical significance was set at P<0.05. Additionally, qualitative descriptors of standardized effects were used such that effect sizes (ES) <0.4, 0.41–0.7 and >0.7 represented small, moderate and large magnitudes of change, respectively [30]. Also, effect sizes were determined using partial eta squared (η_p_^2^) with values of 0.01, 0.06 and above 0.14 represented small, medium and large differences, respectively [30]. Values are expressed as mean ± standard error. Moreover, a one-way ANOVA with repeated measures was used to compare all variables amongst conditioning activities and testing sessions, and revealed that pre-measurements were similar. Then, intra-class coefficient of variations (ICC(2,1)) were calculated and demonstrated moderate to high reliability with values always >0.8 [31], despite the little number of stimulations at each intensity.

## Results

EMG data collected during static stretching were lower than 3% of the EMG recorded during the MVIC (mean: 1.7%, 1.9% and 2.6% for SOL, GM and GL muscles, respectively), which confirms that no muscle contraction occurred during stretching [27]. Regarding the reliability of neurophysiological measures, EMG values recorded during maintained submaximal isometric contractions, in the 200-ms window prior to stimulation, were not significantly different between pre- and post-intervention tests for the MEP, H_max_ and M_max_ variables and for all muscles. Indeed, no significant main or interaction effects were observed for EMG_MEP_, EMG_Hmax_ and EMG_Mmax_ in SOL (P = 0.67, P = 0.22 and P = 0.41, respectively), GM (P = 0.13, P = 0.07 and P = 0.12, respectively) and GL (P = 0.41, P = 0.47 and P = 0.78, respectively).

**Fig 2:**
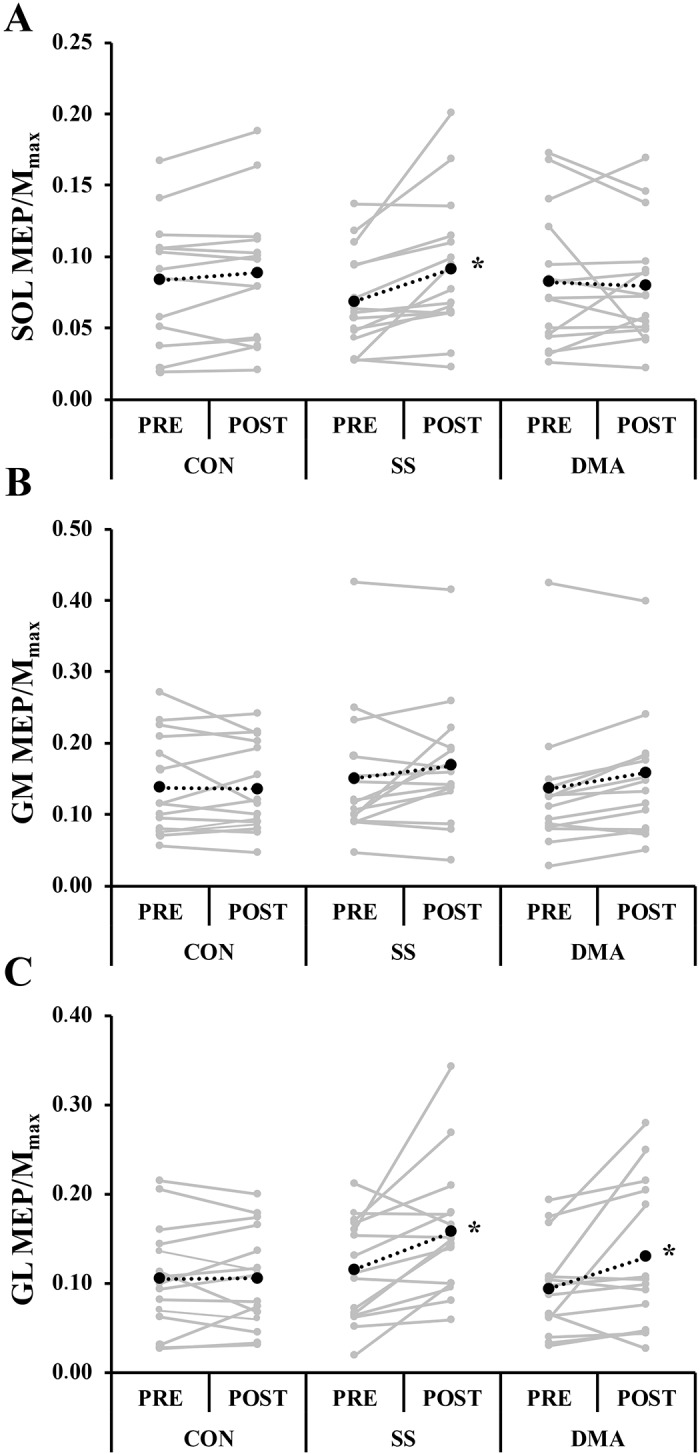
Mean (black dotted lines) and individual (grey lines) values for MEP/Mmax recorded immediately before (PRE) and 10 s after (POST) the three conditioning activities in (A) soleus (SOL), (B) gastrocnemius medialis (GM) and (C) gastrocnemius lateralis (GL). *Significant difference from pre-intervention tests (P<0.05).

No significant main or interaction effects were observed for PTT or MVIT. Voluntary torque developed by plantar flexor muscles during DMA was relatively low (9.04 ± 1.32 Nm) and corresponded to 7.3 ± 3.0% of MVIT. Statistical analysis revealed a significant conditioning activity × time interaction for MEP amplitude and MEP/M_max_ in SOL (P = 0.025, η_p_^2^ = 0.23 and P = 0.028, η_p_^2^ = 0.23 respectively) and GL (P = 0.017, η_p_^2^ = 0.25 and P = 0.026, η_p_^2^ = 0.23, respectively) (Table 1). Regarding GM, a significant main time effect was observed (P<0.001, η_p_^2^ = 0.58 and P = 0.049, η_p_^2^ = 0.23, respectively), with a greater MEP/M_max_ ratio during the post- than the pre-intervention tests. MEP amplitude and MEP/M_max_ were enhanced after SS in SOL (P = 0.001; ES = 0.57 and P = 0.001; ES = 0.54, respectively) and GL (P<0.001; ES = 0.82 and P<0.001; ES = 0.64, respectively) but not GM (Fig 2). Despite no change in MEP amplitude, increase in MEP/M_max_ was also recorded after DMA in GL (P = 0.003; ES = 0.53) but not in SOL or GM. For the three muscles, no significant main or interaction effects were observed for H_max_ and M_max_, amplitudes, H_max_/M_max_ and EMG/M_max_ ratios, or the cortical silent period duration.

**Table 1:** MEP, H_max_, M_max_, MEP/M_max_, Hmax/M_max_, EMG/M_max_ and cSP immediately before (pre) and 10 s after (post) the three conditioning activities for the SOL, GM and GL muscles.

		SOL			GM			GL		
		CON	SS	DMA	CON	SS	DMA	CON	SS	DMA
MEP (mV)	Pre	0.96 ± 0.12	0.77 ± 0.09	1.12 ± 0.17	0.99 ± 0.08	1.07 ± 0.17	1.05 ± 0.12	0.98 ± 0.18	0.88 ± 0.10	0.95 ± 0.14
	Post	1.01 ± 0.13	1.08 ± 0.18 *	1.08 ± 0.16	1.02 ± 0.10	1.13 ± 0.16	1.29 ± 0.15	1.00 ± 0.18	1.28 ± 0.14 *	1.16 ± 0.15
H_max_ (mV)	Pre	7.81 ± 0.73	8.18 ± 0.65	7.91 ± 0.66	2.40 ± 0.27	2.44 ± 0.19	1.96 ± 0.22	2.79 ± 0.28	2.9 ± 0.27	2.15 ± 0.28
	Post	7.32 ± 0.70	7.66 ± 0.69	8.15 ± 0.64	2.02 ± 0.25	2.03 ± 0.22	2.19 ± 0.23	2.75 ± 0.34	2.57 ± 0.27	2.22 ± 0.27
M_max_ (mV)	Pre	10.80 ± 0.68	10.87 ± 0.71	13.69 ± 0.71	9.11 ± 0.97	10.33 ± 1.01	11.00 ± 1.15	13.25 ± 1.09	9.78 ± 0.94	13.77 ± 0.89
	Post	10.18 ± 0.68	12.10 ± 0.69	13.96 ± 0.61	9.62 ± 0.94	12.10 ± 0.69	13.96 ± 0.61	12.75 ± 1.03	9.69 ± 0.89	14.43 ± 0.96
MEP/M_max_	Pre	0.083 ± 0.011	0.069 ± 0.009	0.082 ± 0.012	0.138 ± 0.018	0.150 ± 0.024	0.136 ± 0.024	0.104 ± 0.015	0.115 ± 0.015	0.093 ± 0.013
	Post	0.089 ± 0.012	0.091 ± 0.013 *	0.079 ± 0.011	0.135 ± 0.016	0.168 ± 0.023	0.158 ± 0.023	0.105 ± 0.014	0.157 ± 0.019 *	0.129 ± 0.021 *
H_max_/M_max_	Pre	0.548 ± 0.062	0.594 ± 0.058	0.484 ± 0.051	0.252 ± 0.034	0.287 ± 0.043	0.216 ± 0.038	0.188 ± 0.027	0.218 ± 0.034	0.147 ± 0.026
	Post	0.525 ± 0.068	0.565 ± 0.061	0.508 ± 0.049	0.228 ± 0.035	0.281 ± 0.053	0.213 ± 0.031	0.182 ± 0.029	0.209 ± 0.032	0.204 ± 0.051
EMG/M_max_	Pre	0.018 ± 0.002	0.021 ± 0.002	0.018 ± 0.002	0.039 ± 0.005	0.044 ± 0.005	0.040 ± 0.006	0.029 ± 0.004	0.034 ± 0.004	0.034 ± 0.004
	Post	0.018 ± 0.003	0.021 ± 0.002	0.016 ± 0.001	0.035 ± 0.005	0.051 ± 0.008	0.038 ± 0.005	0.030 ± 0.004	0.037 ± 0.004	0.031 ± 0.005
cSP (ms)	Pre	108.0 ± 5.4	111.4 ± 5.0	109.8 ± 4.8	104.3 ± 5.7	103.6 ± 4.2	104.6 ± 5.2	116.1 ± 10.0	109.3 ± 4.8	109.5 ± 3.6
	Post	107.8 ± 4.6	113.2 ± 5.7	105.8 ± 4.6	104.9 ± 5.5	106.7 ± 5.0	102.8 ± 5.2	107.0 ± 4.0	109.5 ± 5.1	105.2 ± 3.9

Absolute values are expressed as mean ± standard error. Significant difference from:

*pre-intervention tests (P<0.05). In addition, statistical analyses indicate a main effect for time for MEP/M_max_ on GM.

SOL: Soleus; GM: Gastrocnemius medialis; GL: Gastrocnemius lateralis; CON: Control; SS: Static stretching; DMA: Dynamic muscle activity; MEP: Motor evoked potential; EMG: Electromyography; cSP: Cortical silent period

## Discussion

The acute influence of two pre-exercise activities used to condition the neuromuscular system for subsequent exercise has been investigated. The main findings were that (i) static stretching and dynamic muscle activity had no detectible influence on spinal excitability (H_max_/M_max_), but that (ii) corticospinal excitability (MEP/M_max_) was significantly enhanced after both pre-exercise conditioning activities, indicating the presence of facilitation of the corticospinal pathway independent of the type of preceding conditioning intervention.

### Maximal force and activation

Muscle activation, assessed by EMG/M_max_ remained unaltered following static stretching and dynamic muscle activity warm-ups, suggesting that both conditioning activities used in the current study had no detectible effect on descending drive during MVIC. Additionally, voluntary (MVIT) and evoked (PTT) torque remained unaffected. These findings are congruent with others who have used similar stretch or dynamic muscle activity durations and found no change in plantar flexor maximal voluntary isometric torque [32,33].

### Corticospinal excitability

The present findings revealed an ongoing increase in MEP/M_max_ ratio after both pre-exercise conditioning activities, which may reflect a facilitation along the corticospinal tract. It could underpin facilitatory mechanisms located at cortical, spinal and motoneuronal levels [22], and would be primarily related to input from stretch-sensitive afferents [4,14,34] during static stretching and dynamic muscle activity. This ongoing increase is inconsistent with the current literature, which has reported a decrease in MEP amplitude during static stretching itself, which then returned to baseline values as soon as stretching was ended [14,16]. Moreover, a recent study revealed an increase in MEP amplitude up to 2 s following stretching, which then quickly recovered to initial values [17]. The cause of these differences might be attributed to methodological aspects, and especially to the muscle state at the moment of measurements (i.e. relaxed or contracted) [35]. Contrary to these studies whose neurophysiological data were collected in resting muscle, our measurements were performed with a 30% MVIC background contraction. It could be suggested that the influence of muscle stretching on corticospinal pathway modulations would be accentuated when measurements are performed with a background voluntary contraction. In addition, inter-individual variability and/or other methodological aspects [36], such as preceding muscular activity, subject/joint position or stimulation intensity, which differ between these presented researches and the current study, might partly explain these discrepancies. Nevertheless, why MEP was facilitated after static stretching and dynamic muscle activity remains unclear. Budini et al. [17] have suggested that the inhibition related to the stretching would be likely dissipated and possibly counteracted by the opposite movement to reposition the joint to testing position. On another hand, recent findings have suggested that an enhanced MEP amplitude without H-reflex modulation might reflect a transient facilitation at cortical and/or post-synaptic level, to compensate the spinal inhibition induced by muscle stretching [37]. However, our MEP and H-reflex analysis cannot assert the origin of the current modulations. Used together with transcranial magnetic stimulation and neuromuscular electrical stimulation techniques, cervicomedullary stimulation could help define the modulations in the corticospinal pathway, analyzing cervicomedullary motor evoked potential amplitude [38].

With respect to muscle specific modulation, it is notable that MEP/M_max_ was enhanced in both SOL and GL after static stretching, in GL after dynamic muscle activity, and remained unchanged in GM after both modalities. Previous studies have demonstrated that the spinal pathways to SOL and GM motoneuronal pools may differ, resulting in different sensitivity to inhibitory mechanisms [24]; the SOL motoneuron pool receives greater spindle feedback than GM, which might explain, in part, the lack of modulation of corticospinal excitability in GM. However, this speculation remains to be tested by future research.

Our findings also revealed that corticospinal excitability modulations were more pronounced after static stretching relative to dynamic muscle activity; MEP/M_max_ was increased in SOL after static stretching but not after dynamic muscle activity (see Table 1). It can be suggested that the greater ROM required during static stretching relative to dynamic muscle activity might induce more facilitation of the corticospinal pathway, due to a greater amount of sensory afferent feedback (especially from muscle spindles) to the central nervous system. Indeed, it has been shown that corticospinal excitability modulation depends on the amplitude of movement [14,34] with facilitation or inhibition mechanisms being greater with larger amplitude movements. Another possibility is that inputs from joint receptors, which are primarily active toward the end of the range of motion [4,34], modulate corticospinal excitability during static stretching. Although the potential influence of joint receptors is considered to be small relative to the muscle spindles afferents pathway, it cannot be excluded [4,34], especially for large amplitude movements. Finally, it is possible that both conditioning activities elicit different muscle spindles behaviors, and thus differently influence corticospinal excitability, due to the movement pattern itself. It is well known that firing frequency of muscle spindles afferents increases during passive muscle lengthening and decreases during shortening [39,40]. Moreover, it has been demonstrated that firing frequency of primary and secondary afferents respectively decreases and increases when muscle stretch position is maintained [41]. From this basis, the amount of sensory afferent feedback would likely be lower during dynamic muscle activity, which implies cyclic movements, compared to static stretching, which involves static stretch position. Importantly, both pre-exercise activities positively impacted the corticospinal pathway, although some differences may have related to different movement patterns and amplitudes.

Finally, our results revealed that MEP amplitude increases occurred without a change in cortical silent period duration, suggesting that the enhanced corticospinal excitability occurred without change in intracortical inhibition level.

### Spinal excitability

Our data revealed no ongoing effect of static stretching or dynamic muscle activity on spinal excitability. In contrast to our experiments, most muscle stretching studies have evoked H reflexes during the muscle stretch itself and reported H-reflex amplitude to be reduced [13,14, 41]. Post-activation depression appears to be the most plausible hypothesis to explain the H-reflex inhibition during stretching, as well as secondary afferents or post-synaptic inhibitions for longer stretching duration [41]. Recently, the time course of this inhibition has been investigated and highlighted a recovery during stretching time, mostly attributed to a replenishment of neurotransmitter in response to a reduced Ia afferent activity [41]. This mechanism would suggest a complete recovery of the spinal inhibition at the end of the stretching procedure and could partly clarify the current lack of H-reflex changes. As previously explained, another important consideration is that H-reflex measurements have typically been performed in resting muscle, which allows for assessments of change in the basal state but not during muscle contraction when both afferent feedback and supraspinal feedforward traffic will influence H-reflex pathway function [28]. Despite the previous evidence for the decrease in spinal excitability *during* stretching, the current data revealed no change in H-reflex amplitude *after* static stretching, and thus there appeared to be no net change in the function of H-reflex-associated circuitry during muscle contraction. This is in agreement with other studies that have not detected changes in spinal excitability using this measure, even at rest, after a bout of static stretching [5,13,14]. In fact, according to a recent review [42], even when H-reflex depression has been observed as long as 2 s after the removal of a muscle stretch, it certainly appears to be recovered within 15 s. For example, Budini et al. [16] observed an increase in H-reflex after one minute of static stretching, but it returned to baseline values by 40–50 s after stretching. This is consistent with the method adopted in the present study, in which H-reflex was recorded 40–50 s after the conclusion of stretching.

Knowing that factors such as the muscle lengthening velocity [43] or the activation level of the antagonist muscles [13] may influence H-reflex amplitude, we might have expected dynamic muscle activity to elicit a more significant decline in spinal excitability than static stretching. Indeed, H_max_/M_max_ ratio could be influenced by inhibitory contributions from velocity-sensitive receptors such as muscle spindles, whose discharge rate would be increased with the velocity of lengthening [44]. Alternatively, it has been shown that antagonist muscle contraction may result in inhibitory input onto α-motoneurons, reducing H-reflex amplitude [45]. Nevertheless, the present findings were that H_max_/M_max_, measured during isometric contraction, was unaffected after both pre-exercise interventions. It has been suggested that when muscle stretching (i.e. muscle lengthening) is followed immediately by muscle shortening, regardless of the muscle activated (agonist or antagonist) or the contraction intensity, any depression of the H-reflex is removed [42]. This suggests that, when measured during muscular activation after cessation of pre-exercise activity, there was no detectible ongoing alteration of spinal pathway function, independent of activity type, i.e. lengthening velocity, amplitude and muscle activity.

### Study limitations

There are some limitations to the present study that require to be highlighted. First of all, a methodological requirement was to keep the stimulation protocol shorter, in order not to mask any potential transient modulation of corticospinal excitability, which has already shown a rapid tendency to return to baseline [14,16,17]. Therefore, only six MEPs, three H_max_ and three M_max_ were evoked, whilst a greater number of neurophysiological measures are typically considered suitable in order to ensure a high outcome reliability. Nonetheless, ICC demonstrated moderate to high reliability. In addition, the study design did not allow to determine whether spinal modulation occurred when MEP amplitude was increased (i.e. between 10 and 35 s after conditioning activity). Indeed, neurophysiological measures were always performed in the same order that entailed therefore a duration (40–50 s) between the end of conditioning activity and the H-reflex recordings likely too long to observe any spinal inhibition, which appears to recover within 15 s after stretching in the literature [42]. In the same way, knowing the fast recovery of stretch-induced MEP modulations [17], the time from the end of stretching procedure and the last stimulation has to be considered in the results interpretation. Indeed, averaging stimulations over this time period (35 s in the present study) does not exactly reflect the modulations which likely occur immediately after stretching.

## Conclusion

Both static and dynamic muscle activity induced similar increases in corticospinal excitability without affecting muscle function. The lack of decrement in maximal voluntary and evoked force production after static stretching is important, since it indicates that function was retained. Furthermore, while this study provides new insights into the corticospinal pathway changes following static stretching and dynamic muscle activity, the mechanisms underpinning the increase in corticospinal excitability are still unclear.
